# Advancing Antimicrobial Stewardship in Canadian Dentistry: Early Insights Into a Toolkit to De-implement Overprescribing

**DOI:** 10.1016/j.identj.2026.109618

**Published:** 2026-05-23

**Authors:** Christiana Martine, Susan Sutherland, Caroline Fulop, Karen Born, Sonica Singhal

**Affiliations:** a Faculty of Dentistry, University of Toronto, Toronto, Canada; b Ottawa Public Health, The Ottawa Hospital, Ottawa, Canada; c Institute of Health Policy, Management and Evaluation, Dalla Lana School of Public Health, University of Toronto, Toronto, Canada

**Keywords:** Antimicrobial stewardship, Dentistry, Antibiotic prescribing, Antimicrobial resistance, De-implementation, Qualitative research

## Abstract

**Introduction and Aims:**

Despite dentistry’s contribution to antibiotic use and antimicrobial resistance, antimicrobial stewardship in Canadian dentistry remains at an early stage. As strategies focused on adherence to evidence-based care have shown limited success in changing prescribing behaviour, the focus has shifted to the de-implementation of low-value care. This study explored dentists’ perspectives on an antibiotic prescribing toolkit and examined how it may support de-implementation.

**Methods:**

An exploratory qualitative study employed focus groups with 16 dentists who attended toolkit webinars. Dentists who had not downloaded the toolkit were excluded. Discussions were recorded, transcribed, and analysed inductively. Findings were discussed in relation to stewardship and de-implementation research.

**Results and Discussion:**

The analysis yielded 5 themes—clarifying a key message, reminding and providing a talking point, supporting difficult conversations, legitimising nonprescribing, and moving beyond “preaching to the choir”—that align with de-implementation strategies in dentistry and other health fields, including unlearning, extinction cues, substitution, counterconditioning, and champions. Participants highlighted the need for concomitant interventions, such as audit and feedback, education, software integration, and regulatory and professional engagement, to support de-implementation, consistent with the de-implementation literature.

**Conclusion:**

The toolkit shows potential to support the de-implementation of unnecessary dental antibiotic prescribing by introducing mechanisms that reduce reliance on low-value antibiotic use. Feedback suggests the toolkit helps shift outdated prescribing habits, manage patient expectations, and legitimise nonprescribing decisions. However, meaningful progress will require multilevel strategies. Future research should evaluate, using quantitative methods, whether the toolkit influences prescribing behaviours longitudinally, as well as whether its integration within broader stewardship interventions supports sustained de-implementation.

**Clinical Relevance:**

This study identifies strategies to support responsible antibiotic use in dental practice. The findings show how the toolkit may serve as a practice-based resource by offering communication tools, reminders, and substitution strategies that help dentists manage patients without antibiotics, while reducing patient expectations and dentists’ concerns about not prescribing.

## Introduction

Antimicrobial stewardship (AMS) in Canadian dentistry remains at an early stage of development. Antimicrobial resistance (AMR) has been identified by the World Health Organization as 1 of the 10 major threats to population health globally.^1^ In 2019, it was associated with nearly 5 million deaths and directly responsible for 1.27 million deaths.^2^ As dentistry accounts for approximately 10% of all antibiotics prescribed worldwide^3^ and 11.8% of antimicrobial prescriptions in Canada in 2023,^4^ it has a critical role to play in addressing this escalating global health threat. Strengthening AMS in dental prescribing is therefore essential to bring dentistry into alignment with national and international AMR efforts aimed at curbing unnecessary antibiotic use.

Although several drivers of antibiotic overuse in Canadian dentistry have been identified,^5^ research also suggests that merely advising prescribers to follow updated recommendations rarely changes behaviour.^6^ Efforts to reduce antibiotic prescribing across a wide range of prescribers show that this is challenging due to a complex set of factors such as habits, culture, perceived patient expectations, information asymmetry, and limited time during clinical interactions.^7^^,^^8^ Evidence-based interventions are often poorly implemented and fail to reach those they are intended to support.^9^

While implementation science has traditionally focused solely on increasing adherence to evidence-based care,^6^ attention is increasingly shifting towards stopping—or de-implementing—forms of low-value care,^10^ which, in the context of unnecessary prescribing, can be defined as practices in which the potential harms exceed the expected benefits.^11^ De-implementation science seeks to identify low-value practices, understand the factors that sustain them, and implement evidence-based interventions to eliminate them.^6^ While the de-implementation of antibiotic prescribing has been extensively researched in medicine,^11^ there is a lack of such studies in dentistry, where it remains “an emerging and underexplored field.”^12^

The aim of this study is to examine how a dental antibiotic prescribing toolkit may support the de-implementation of unnecessary prescribing in dentistry and to explore how dentists’ early reactions to it may align with findings from the broader de-implementation literature. In addition, it identifies strategies for advancing de-implementation efforts moving forward. These findings may help to advance antimicrobial stewardship within Canadian dentistry and may also offer insights for dental policy and practice within international efforts to strengthen antimicrobial stewardship.

## Toolkit development and earlier AMS initiatives

To assess stakeholders’ perspectives on the main drivers of dental antibiotic overuse and potential AMS strategies, our team at the University of Toronto conducted a series of focus group discussions with key Canadian health care leaders and AMS experts during the summer of 2023. The analysis of these discussions culminated in a qualitative study that identified 3 main drivers of antibiotic overprescribing in dentistry: outdated prescribing patterns, fear and risk aversion, and the use of antibiotics as a Band-Aid.^5^ The study’s findings informed a subsequent workshop held as part of a tripartite collaboration among researchers from the Universities of Toronto, Manchester, and Melbourne. The workshop brought AMS stakeholders together to outline a more structured and coordinated approach to strengthening dental AMS. Priorities identified included the collection of robust data, the development of improved accountability metrics for antimicrobial prescribing, and the creation of improved clinical guidelines to support appropriate antibiotic use, recognised as an essential first step in strengthening AMS practices in Canadian dentistry.^13^

In 2024, in response to these priorities and to initiate practical efforts to advance dental AMS, the Choosing Wisely Canada (CWC) toolkit, “Taking the Bite Out of Tooth Pain: A Toolkit on Using Antibiotics Wisely for Managing Tooth Pain in Adults”^14^ (Appendix A; key features in Box 1), was developed through a partnership between experts in AMR and AMS, involving University of Toronto academics, the Canadian Dental Association, the Royal College of Dental Surgeons of Ontario, the College of Family Physicians of Canada, and CWC (see toolkit, p. 13, for the full list of collaborators).

**Table:** **Box 1.** Highlights of “Taking the Bite out of Tooth Pain: A Toolkit on Using Antibiotics Wisely for Managing Tooth Pain in Adults”

• **Interprofessional applicability:** designed for dentists, physicians, and other health care providers managing adult patients with tooth pain in diverse settings • **Clear evidence-based message:** antibiotics do not cure most causes of tooth pain and should be reserved for cases with systemic involvement • **Standardised management of tooth pain:** structured approaches for dental and nondental settings **• Antibiotic of choice for cases with systemic involvement:** recommends first-line agents, dosing, duration, and alternatives tailored to allergy history • **Tooth pain prescription:** provides a non-antibiotic prescription focused on managing symptoms through analgesics, self-care strategies, and follow-up instructions directing patients to seek definitive dental treatment • **Poster and screensaver:** serve as reminders for providers to help prevent relapse into outdated prescribing habits while reducing patient expectations of receiving an antibiotic during the appointment • **Frequently asked questions (FAQs):** offers additional clinician and patient resources explaining indications for antibiotics, prophylaxis considerations, penicillin allergy, and medically complex cases • **Quality improvement supports:** provides strategies to evaluate the appropriateness and effectiveness of antibiotic prescribing in clinical practice

Alt-text: Unlabelled box dummy alt text

The toolkit, designed to support the de-implementation of unnecessary antibiotic prescribing not only among dentists but also among other health care providers who see patients for toothaches, was created using an established approach employed by CWC for antimicrobial stewardship resources, including initiatives such as *The Cold Standard* for respiratory tract infections. An expert working group was convened, including dentists and a family physician with experience in developing CWC resources, the lead infectious diseases physician for the Using Antibiotics Wisely campaign, quality improvement specialists with expertise in implementation science, and representatives from dental regulatory bodies and the national dental association. Development was guided by key criteria used by CWC for toolkit design, including evidence-informed content to support standardised decision-making and reduce clinical uncertainty; tools that address behavioural drivers and enhance communication to manage patient expectations; and resources that can be readily integrated into the workflow and electronic medical record systems of busy clinical practices. The toolkit underwent peer review prior to its release.

Among other features aimed at reducing emphasis on antibiotics, the toolkit is innovative in the Canadian dental setting as it includes treatment algorithms for different practice settings, a non-antibiotic prescription focused on managing symptoms through analgesics and self-care strategies, a concise table of recommended antibiotics, a tool for rapidly assessing penicillin allergy, frequently asked questions for patients and providers, and a “toothache” prescription for symptom management. In their digital format, the resources can be downloaded, displayed, or shared with patients to reinforce clinician messaging before, during, and after appointments. All resources are hyperlinked for easy navigation, and the toolkit can be integrated into electronic patient records.

## Toolkit launch: early practitioner reactions

Following the toolkit’s launch, we explored dentists’ early reactions to this resource and identified practical approaches to support dissemination and adoption.

### Methodology

We conducted an exploratory qualitative study using focus group discussions as the data collection method. Following approval by the University of Toronto’s Research Ethics Board (Protocol No. 00048146; approved July 15, 2025), dentists who attended 1 of 3 online webinars on the toolkit, held following its launch and hosted by the Royal College of Dental Surgeons of Ontario, the American College of Dentists (Ontario Section), and the Canadian Dental Protective Association, were invited by email to participate in the discussions. Dentists who had not downloaded the toolkit from the CWC website were excluded. A total of 17 dentists met the inclusion criteria and provided written informed consent; however, only 16 eventually participated, as 1 participant was unable to access the link to the virtual meeting at the scheduled time. Three focus groups (with group sizes of n = 5, 6, and 5 participants) were conducted via Zoom for approximately 60 minutes each using an in-depth, semistructured interview guide (Appendix B). Data were transcribed verbatim using Zoom’s audio transcription and Otter AI, then proofread and coded line by line manually by researcher C.M. using a semantic approach. Data were analysed inductively following Braun and Clarke’s thematic framework^15^ and conducted iteratively through repeated review of the raw data to deepen understanding and ensure that interpretations remained consistent with the original data and research questions. Codes and preliminary categories were reviewed and refined with researchers S.Si., S.Su., K.B., and C.F. at a later stage. Results were subsequently further developed in collaboration with the team to enhance trustworthiness.^16^ Reflexive discussions were held among the research team throughout analysis.

## Results and discussion

### Understanding early practitioner responses in the context of de-implementation mechanisms

Dentists’ early perceptions of the toolkit are presented below and organised into 5 themes (see Table 1 for an overview of themes and brief descriptions). These themes were then contextualised in relation to antimicrobial stewardship and the de-implementation literature in both medicine and dentistry.

**Table 1:** Dentists’ early perceptions of “Taking the Bite Out of Tooth Pain: A Toolkit on Using Antibiotics Wisely for Managing Tooth Pain in Adults.”

Theme	Description
Clarifying a key message	The toolkit was found to be useful and easy to use, providing a clear, evidence-based message that antibiotics do not cure most causes of tooth-related pain, which was viewed as important for educating providers, dental staff, and patients.
Reminding and providing a talking point	The toolkit poster, available in print and as a digital screensaver, was valued for maintaining visibility of key messages, serving as a reminder for clinicians and a conversation starter for patients in clinical and waiting-room settings.
Supporting difficult conversations	The toolkit was found to serve as a communication aid, in that it provided clinicians with tangible alternatives, helping them to navigate challenging patient conversations, potentially reducing reliance on prescribing.
Legitimising nonprescribing of antibiotics	The toolkit was perceived to offer reassurance and formal support for decisions not to prescribe antibiotics, helping to reduce fear of negative repercussions and strengthening confidence in nonprescribing decisions.
Moving beyond “preaching to the choir”	A need for targeted dissemination strategies was identified to engage practitioners who are not yet attuned to antimicrobial stewardship, highlighting education, accountability mechanisms, and antimicrobial stewardship champions as key opportunities.

#### Clarifying a key message

Overall, participants viewed the toolkit favourably, describing it as “useful” and easy to use. Its key strength lies in communicating a clear, evidence-based message: antibiotics will not cure most causes of tooth-related pain. In the context of de-implementation science, and drawing on behavioural mechanisms from classical conditioning,^6^ this central message can be interpreted as supporting the new learning necessary to replace entrenched prescribing habits, thus addressing a key driver of antibiotic overuse in dentistry.^5^ This process appears consistent with the cognitive “unlearning” pathway described in the de-implementation model by Helfrich et al,^17^ whereby providers are prompted to reassess outdated beliefs and adjust their behaviour in response to new evidence. Participants in the focus group emphasised that the toolkit’s clear and straightforward presentation of new evidence is critical not only for changing providers’ long-held assumptions but also for patients and dental staff, including front desk personnel, hygienists, and dental assistants. They noted that educating these team members is essential. For instance, receptionists, who are often the first point of contact, can help to shape patient expectations, a factor known to contribute to antibiotic overuse in dentistry,^5^ even before the consultation. Without being exposed to this new information, staff may unwittingly reinforce these expectations.

The main thing [advantage] is that, well, one of the many main things—we’ll call them in the toolkit—was that an antibiotic will not cure, quote, unquote, a toothache. And that’s invaluable information when it lists what an antibiotic can and can’t do, and I very much like that. (Participant 7)

#### Reminding and providing a talking point

Available both as a printable version and as a digital screensaver, the toolkit poster was consistently identified by dentists as a particularly valued feature. Participants noted that the digital screensaver option helps to maintain continuous visibility of the key message that “antibiotics won’t cure most toothaches,” which may promote new learning through repeated exposure. In this way, it may function as an extinction cue at the point of care, a de-implementation strategy that can serve as a reminder of the provider’s commitment to new learning, thus avoiding relapse of outdated prescribing habits.^6^ Moreover, given that it can be displayed and made visible in waiting areas, as participants observed, it can arguably also function as a visual cue for patients and as a “talking point” for dentists that encourages reflection before the appointment begins. In addition, participants noted that the poster’s visibility in waiting areas may help to manage patient expectations, a known driver of unnecessary prescribing in dentistry.^5^

It’s a great poster . . . So that is there as a talking point. If someone starts trying to argue or ask [about] it—and even just something like that while they’re waiting, they might ask about—that’s a kind of an introduction, and it’s a reminder to our clinicians as well. Hey! Maybe think before [you prescribe an antibiotic], you know. (Participant 4)

#### Supporting difficult conversations

Participants noted that the toolkit serves as a communication aid for providers navigating, as one dentist put it, “difficult conversations with patients” who arrive at their appointments expecting an antibiotic prescription. By equipping dentists with key points to substantiate their discussions with patients, the toolkit provides alternative actions that enable providers to “do something” other than prescribe. When considering the toolkit’s “Tooth Pain Prescription” sheet, for instance, it provides a tangible alternative that can replace an antibiotic prescription, offering a concrete action for providers during the clinical encounter.

You know, [the toolkit helps with] difficult conversations which are obviously difficult to have, especially with patients. Some can, and some are not as bold to have those conversations with patients, and so they give in, whether it’s antibiotics or pain medications. (Participant 1)

From a de-implementation perspective, this suggests alignment with substitution, a strategy in which a low-value behaviour is replaced with an alternative action,^6^ to address another prescribing driver, namely, the provider’s need to “do something”^5^ for a patient in pain during a consultation. This substitution mechanism is also presented in the dual model for de-implementation by Helfrich et al,^17^ which characterises it as “replace or displace the ineffective practice.”

#### Legitimising nonprescribing of antibiotics

Dentists highlighted that having a formal resource to reference at the point of care helps them feel supported in not prescribing antibiotics, easing concerns about potential negative repercussions and strengthening providers’ confidence in adopting a new pattern of treatment. By reducing fear of negative consequences associated with nonprescribing (eg, patient infection, college reprimands),^5^ the toolkit helps to reinforce the newly adopted behaviour and prevent relapse into outdated prescribing habits. In this way, the toolkit is consistent with a counterconditioning approach, reinforcing newly learned behaviours and strengthening the likelihood of de-implementation.^6^ The clear and printed information presented in the toolkit legitimises nonprescribing decisions, reassuring dentists that their clinical choices align with current evidence while, at the same time, signalling to patients that care is guided by the most up-to-date recommendations.

When I’m doing a case presentation discussing these options with the parents, what will happen is, “oh, I’d like an antibiotic.” Mmmm, well, no. In this case, you don’t need it, and here is why. And that’s where the toolkit gives me hard data to back me up. (Participant 8)

#### Moving beyond “preaching to the choir”

However, to achieve the toolkit’s potential to support changes in practice through these mechanisms, it is critical to create a coordinated dissemination strategy for its uptake, particularly given evidence showing that the implementation of guidelines in dentistry remains a challenge, with one of the lowest rates of guideline compliance among health care professions.^18^ A major challenge to the toolkit uptake identified by dentists was the difficulty of reaching and engaging practitioners who are not yet attuned to this issue. As one focus group participant noted, “We need to talk to the people who are not here. That’s the blind spot. Mhmm. . . . For sure. Yeah. ‘Preaching to the choir tonight.’ They’ll know their barriers better than we would” (participant 8). Dentists also highlighted several opportunities to address this barrier, including strengthening education from the ground up in dental schools, enhancing continuing education through mandatory or credit-bearing options, ensuring repeated AMR and AMS messaging, establishing stronger accountability mechanisms through engagement with regulatory colleges and professional associations, and creating AMS champions within dentistry. These suggestions align with established dissemination and implementation strategies in oral care.^9^ Champions, for instance—fellow clinicians who have demonstrated commitment to stewardship efforts—have been recommended in dental care and other settings as a strategy to support the de-implementation of low-value care.^9^^,^^19^

While the toolkit supports behavioural shifts through such mechanisms, real change will likely require additional interventions implemented alongside it. The next section outlines such interventions, drawing on both the broader and dental-specific literature, as well as insights from the focus group discussions.

### Advancing de-implementation through multifaceted interventions

In the dual-pathway model, proposed by Helfrich et al,^17^ “unlearning” reflects a conscious, intentional process of evaluating new evidence and changing behaviour, and “substitution” involves an unconscious, automatic response. This model is also useful for providing a heuristic frame for considering additional de-implementation tools to reinforce behavioural change in the dental arena.

An audit and feedback approach, wherein clinicians receive performance data benchmarked against their peers and engage in follow-up reviews of progress, is a well-established strategy in the primary care setting^20^ and can serve as a practical method to further operationalise deeper and more sustained unlearning of entrenched behaviours by encouraging reflection on practice.^17^ In addition, the inclusion of information that explicitly communicates the serious deleterious effects of overprescribing alongside comparative prescribing feedback, similar to an audit feedback program recommended in the context of antipsychotic medications,^17^ would align with focus group participants’ observations that the consequences of antimicrobial resistance need to be clearly communicated. Echoing our earlier qualitative finding in which AMR was likened to “climate change,”^5^ dentists highlighted that it often feels distant from everyday dental practice and, as such, requires stronger emphasis.

Although a study in the Netherlands identified dentists’ fear of performance monitoring and the use of data as potential barriers to improving oral health care,^21^ evidence from other dental settings supports the potential of audit and feedback to influence prescribing behaviour. For instance, an audit and feedback program in the context of dental primary care in the United Kingdom’s National Health Service was shown to significantly reduce antibiotic prescribing among dentists.^22^ However, evidence from other studies, including a systematic review evaluating strategies used to implement clinical guidelines in dental practice, suggests that the impact of audit and feedback on guideline adherence, when delivered as a single intervention, has been variable in other dental care settings^9^^,^^18^ with similar inconsistent findings in the broader health care research.^18^

Such variability reflects a broader pattern in the implementation literature: multifaceted interventions tend to be more effective than individual strategies. This can arguably be attributed to the fact that barriers to improving dental care operate across multiple levels, from individual awareness to policy development, highlighting the need for a coordinated approach.^23^ An overview of 46 systematic reviews, many of which focused on reducing unnecessary antibiotic prescribing, found that combined approaches outperformed individual interventions.^11^ A subsequent meta-analysis corroborated this pattern, with multilevel interventions, particularly those combining provider and patient education with audit and feedback, achieving substantially larger reductions in low-value care than provider education or audit and feedback alone.^24^

Similar to findings in other health care settings, evidence in dentistry indicates that audit and feedback are more effective when combined with complementary implementation strategies than when used on their own.^18^^,^^25^ For instance, in a prospective cohort study involving 15 private practice dentists, the combination of baseline knowledge assessment, education, individualised audit and feedback, and follow-up interventions increased the appropriateness of treatment-related antibiotic use from 15% to 90.2%.^25^

Focus group participants also suggested integrating the toolkit’s messaging into dental practice management software and electronic record systems**.** According to Helfrich et al,^17^ such electronic prompts can help to support automatic cognition by modifying the environmental cues that trigger habitual prescribing, disrupting the tendency to rely on ingrained patterns. In dentistry, evidence similarly points to the perceived value of embedding antibiotic decision support tools within dental electronic record systems, including commonly used dental platforms such as axiUm.^26^ This perspective was reflected in one of the focus group discussions, with one participant noting that when these tools are inserted “into your computer software, so that when you prescribe an antibiotic, you’re prompted, you know.” This observation reflects the fact that many instances of low-value prescribing stem from routine, automatic workflows rather than deliberate clinical reasoning; hence, embedding updated guidance, for instance, within dental electronic record systems can reshape the choice architecture and replace these automatic responses with more appropriate alternatives, further strengthening the “substitution” mechanism for de-implementation.^17^

Dentists also highlighted the need for greater engagement from both regulatory colleges and professional associations to reinforce appropriate prescribing through greater accountability. This aligns with the view by Helfrich et al^17^ that altering the work environment—here, through professional bodies’ policies—can support substitution by limiting low-value options and steering clinicians towards evidence-based care. Examples raised by participants included assessing newly graduated and internationally trained dentists’ knowledge of antimicrobial stewardship through Canada’s licensing process, stronger dissemination of stewardship messages through professional associations, and increased integration of AMS content within dental education and continuing professional development—supports that, while not constituting substitution mechanisms in themselves, help create the conditions in which substitution can take hold.

### Limitations

A limitation of this study is that dentists who had attended the webinar and downloaded the toolkit may represent a group already more attuned to antimicrobial stewardship, potentially introducing selection bias and limiting insights to practitioners who may be less engaged but could benefit most from such interventions. This may also limit the transferability of findings to similar contexts. In addition, given the exploratory nature of the study, the findings are based on early perceptions only, and behavioural change was not directly measured. Nevertheless, this study provides early insights into the use of the toolkit as a de-implementation tool, identifying potential mechanisms and suggesting considerations for advancing antimicrobial stewardship in dental practice.

## Conclusion

The “Taking the Bite Out of Tooth Pain: A Toolkit on Using Antibiotics Wisely for Managing Tooth Pain in Adults” can be considered a first step towards the de-implementation of unnecessary antibiotic prescribing in Canadian dentistry. This study identifies mechanisms that may support changes in behaviour and reduce reliance on low-value antibiotic use. Although behavioural change was not directly measured, early practitioner feedback suggests that the toolkit may support changes in outdated prescribing habits, help manage patient expectations, and legitimise nonprescribing decisions, reflecting key de-implementation mechanisms. However, meaningful progress will require multilevel strategies—such as provider and patient education, audit and feedback, integration into electronic dental records, and regulatory supports—to advance de-implementation of dental antibiotic overprescription. Future research should evaluate, using quantitative methods, whether the toolkit influences prescribing behaviours longitudinally, as well as whether its integration within broader stewardship interventions supports sustained de-implementation. As countries seek to align dentistry with global efforts to curb antimicrobial resistance, these early insights highlight the need for coordinated, multilevel strategies to reduce unnecessary antibiotic use and embed stewardship as a core element of dental care.

## Author contributions

C.M.: Conceptualisation, methodology, data collection, formal analysis (manual coding and thematic analysis), and writing – original draft. S.Su.: Conceptualisation, data collection, formal analysis (coding review and thematic refinement), writing – review and editing. S.Si.: Conceptualisation, data collection, formal analysis (coding review and thematic refinement), writing – review and editing. C.F.: Data collection, formal analysis (coding review), writing – review and editing. K.B.: Conceptualisation, methodology, data collection, formal analysis (coding review and thematic refinement), writing – review and editing. All authors have approved the manuscript and agree with its submission to the *International Dental Journal*.

## Funding

This study was supported by Choosing Wisely Canada. Although Choosing Wisely Canada contributed to the development of the toolkit explored in this study, it had no role in the qualitative study design, data collection, analysis, interpretation, or writing of the manuscript.

## Conflict of interest

None disclosed.

## Appendix Supplementary materials

Image, application 1

Image, application 2

## Footnotes

Supplementary material associated with this article can be found in the online version at doi:10.1016/j.identj.2026.109618.
