# Supplementary material for: Advancing Antimicrobial Stewardship in Canadian Dentistry: Early Insights Into a Toolkit to De-implement Overprescribing
Source: Int Dent J. 2026 May 23;76(4):109618. doi: 10.1016/j.identj.2026.109618 (PMC13226238; doi:10.1016/j.identj.2026.109618)
Supplement: Supplementary file 2 [file mmc2.docx]

# Appendix B - Interview Guide

| **Introduction**  Thank you for joining us today to share your perspectives on the new toolkit developed to assist dentists, physicians, and other health professionals in prescribing antibiotics for adults presenting with tooth pain.  AMR has been described as a global crisis, currently causing 700,000 deaths a year, with this number increasing steadily. Overprescribing of antibiotics in health care is a contributing factor. Dentists prescribe about 10% of antibiotics in Canada and elsewhere, and it is estimated that up to 80% of these may be unnecessary.  Our earlier research suggested that dentists want resources to use in their discussions with patients about why antibiotics should not be prescribed unnecessarily. Also, how to handle the challenging condition of severe toothache, where antibiotics are expected by patients and prescribed by both dentists and physicians. This led us, in partnership with Choosing Wisely Canada, to develop the toolkit *Taking a Bite Out of Tooth Pain: A Toolkit for Using Antibiotics Wisely for Managing Tooth Pain in Healthy Adults for Healthcare Providers*.  In our discussions today, we aim to understand your perspectives on the toolkit, including any barriers and challenges to its implementation. This will help us develop strategies for more effective toolkit dissemination and implementation**.**  **Before we begin, we would like to remind you of your rights as a participant in this study. Your participation is entirely voluntary, and you may withdraw at any time without any consequences. We would also like to remind you to respect the confidentiality of what others say and ask that you do not discuss what was said during the meeting outside of this setting. You may also refuse to answer any questions you do not wish to answer and still remain in the study. You may withdraw your consent and discontinue participation without penalty. Before we commence the discussion, please feel free to ask any questions or share any potential concerns that you might have. Does anyone have anything they'd like to add or say before we begin?**  To begin, could you tell me a little bit about why you decided to participate in the webinar and how, if at all, it relates to your interest in dental antimicrobial stewardship (AMS)? | |
| --- | --- |
| **Question Domains** | |
| **Initial perception of the toolkit** | 1. What was your general impression of the toolkit created to assist dentists, physicians, and other health professionals in prescribing antibiotics for adults presenting with tooth pain?   *Probes*   - *How user-friendly did you find the toolkit for guiding the prescribing of antibiotics?* - *Do you feel the toolkit effectively addresses all of your needs when prescribing antibiotics for adults presenting with toothache?* - *Which parts of the toolkit do you believe will be most helpful for your practice? Why?* - *Is there any part of the toolkit that you found unclear? If so, how can it be improved?* |
| **Potential effectiveness and impact on decision-making** | 1. Do you feel that the presentation of the toolkit influenced your decisions related to prescribing antimicrobials?   *Probes*   - *If so, in which instances? Are there any situations where you would choose not to follow the toolkit?* - *How comfortable are you with using the toolkit?* - *Are there any areas where you would hesitate to use the toolkit? Why?* - *For those of you who have started using the toolkit, what has been your experience so far?*   *- Were there any obstacles/barriers to adopting it in your practice?*  *- How did you implement it (e.g., print-outs, meetings with staff, etc.)?*  *- How many people in your practice are using the toolkit, and what are their roles?* |
| **Implementation/**  **Dissemination** | 1. What are some of the opportunities to improve the implementation of the toolkit in individual practices?   *Probes*   - *What are some of the challenges?* - *How helpful are mandatory continuing education courses in nudging dentists into adopting the toolkit?* - *Is additional education and/or training needed to support its implementation?* - *How can we better support dentists in using the toolkit?* |
|  | 4. What are some opportunities to promote the widespread implementation of the toolkit?  *Probes*   - *What are some opportunities related to the dissemination of the toolkit?* - *Any challenges?* - *How can dental organizations help, if at all, in this kind of knowledge dissemination? Should their role be focused on guidance, enforcement, or perhaps both?* |
| **Conclusion/Summary remarks** | Is there anything you would like to mention about the toolkit’s usage, implementation, or dissemination that we have not talked about today?   - Any final thoughts or suggestions? |
| **Debrief** | |
